# A Ca^2+^ sensor BraCBL1.2 involves in BraCRa-mediated clubroot resistance in Chinese cabbage

**DOI:** 10.1093/hr/uhad261

**Published:** 2023-12-13

**Authors:** Yinglan Piao, Shizhen Li, Yiduo Chen, Sisi Zhao, Zhongyun Piao, Haiping Wang

**Affiliations:** State Key Laboratory of Vegetable Biobreeding, Institute of Vegetables and Flowers, Chinese Academy of Agricultural Sciences, Beijing 100081, China; State Key Laboratory of Plant Cell and Chromosome Engineering, Institute of Genetics and Developmental Biology, Chinese Academy of Sciences, Beijing 100101, China; Institut für Biologie und Biotechnologie der Pflanzen, Westfälische Wilhelms-Universität, Münster 48143, Germany; State Key Laboratory of Vegetable Biobreeding, Institute of Vegetables and Flowers, Chinese Academy of Agricultural Sciences, Beijing 100081, China; College of Horticulture, Shenyang Agricultural University, Shenyang 110866, China; State Key Laboratory of Vegetable Biobreeding, Institute of Vegetables and Flowers, Chinese Academy of Agricultural Sciences, Beijing 100081, China

## Abstract

Clubroot disease caused by *Plasmodiophora brassicae* (*P. brassicae*) severely threatens the cultivation of Cruciferous plants, especially Chinese cabbage. Recently, resistance genes in plants have been reported to encode for a Ca^2+^-permeable channel in the plasma membrane, which can mediate the cytosolic Ca^2+^ increase in plant cells upon pathogen attack. However, the downstream Ca^2+^ sensor and decoder are still unknown. In this study, we identified the virulent and avirulent *P. brassicae* isolates (Pbs) of two near isogenic lines, CR 3–2 and CS 3–2, with CR 3–2 harboring clubroot resistant gene *BraCRa*. The transcriptomic analysis was then conducted with CR 3–2 after inoculating with virulent isolate PbE and avirulent isolate Pb4. From the differentially expressed genes of transcriptomic data, we identified a Ca^2+^-sensor encoding gene, *BraCBL1*.*2*, that was highly induced in CR 3–2 during infection by Pb4 but not by PbE. Moreover, GUS histochemical staining and subcellular localization analysis revealed that *BraCBL1*.*2* was specifically expressed in the root hair cells of *Arabidopsis* and encoded a putative Ca^2+^ sensor localized in the plasma membrane. We also developed an assay to investigate the BraCRa-mediated hypersensitive response (HR) in tobacco leaves. The results suggest that BraCBL1.2 is involved in the BraCRa-mediated plant ETI immune response against *P. brassicae*. In addition, we verified that overexpression of *BraCBL1.2* enhanced clubroot resistance in *Arabidopsis*. Collectively, our data identified the involvement of a Ca^2+^ sensor in BraCRa-mediated clubroot resistance in Chinese cabbage, providing a theoretical basis for further research on the resistance of Chinese cabbage to *P. brassicae*.

## Introduction

Clubroot is a soil-borne disease that poses a threat to the production of Brassicaceae crops worldwide, especially Chinese cabbage (*Brassica rapa* L. ssp *pekinensis*) [1, 2]. Clubroot is caused by the obligate biotrophic protist *Plasmodiophora brassicae*, which belongs to the plasmodiophorids in the eukaryotic kingdom of Rhizaria [3]. In agricultural production, planting disease-resistant materials is the most effective method for preventing and controlling clubroot disease [4]. Therefore, identifying resistance genes and deciphering the mechanism of resistance is the premise and foundation for conducting disease-resistance breeding and is the key to solving production problems.

Plants are equipped with two layers of the innate immune system: pathogen-associated molecular pattern (PAMP)-triggered immunity (PTI) and effector-triggered immunity (ETI) [5]. Together, these defense mechanisms enable plants to resist pathogenic attack and promote their survival in their stationary habitat [5]. Compared to PTI, ETI mediated by plant resistance proteins (R proteins) is more specific to pathogens and trigger a hypersensitive reaction (HR) [6]. Most plant R proteins consist of nucleotide-binding site (NBS) and leucine-rich repeat (LRR) domains, collectively known as NB-LRR (NLR) proteins [7]. Based on their amino-terminal structural features, NB-LRR proteins can be further categorized into two types: Toll/interleukin-1 receptor (TIR)-NB-LRR (TNL) proteins and coiled-coil NB-LRR (CNL) proteins [7]. To date, two cloned clubroot resistance genes, *BraCRa* and *Crr1a*, which confer resistance to *P. brassicae*, belong to TNL [8, 9].

Calcium ions (Ca^2+^), as ubiquitous second messengers in plants, are implicated in both PTI and ETI responses [10, 11]. In ETI, various plant R proteins can form Ca^2+^ channels on the plasma membrane to generate cytoplasmic Ca^2+^ signals [7, 12]. Recently, it was reported that *WeiTsing*, a newly identified endoplasmic reticulum localized Ca^2+^ channel was expressed in the pericycle of *Arabidopsis* and confers resistance of Brassicaceae plants to clubroot [13]. However, none of the downstream Ca^2+^ sensors are known to be involved in clubroot resistance in *B. rapa*. In plants, there are two types of Ca^2+^ receptors, namely sensor responders and sensor relays [14, 15]. The Ca^2+^-dependent protein kinase (CDPK) family belongs to the sensor responders, while calmodulin (CaM), calmodulin-like proteins (CMLs), and calcineurin B-like (CBL) proteins belong to the sensor relays. A total of 127 homologs of the *CDPK* supergene family, 17 homologs of *CBLs*, and 80 homologs of *CaM* and *CMLs* have been identified in *B. rapa*[16–18]. Growing evidence indicates these Ca^2+^ receptors are widely implicated in deciphering immune-mediated calcium signaling and initiating subsequent immune defense responses [12, 13, 19–22].

Generally, upon binding to calcium ions, AtCBLs activate specific downstream CBL-interacting protein kinases (CIPKs) by relieving their self-inhibition function through interaction [10]. A plethora of studies have demonstrated that the CBL-CIPK signaling pathway is widely involved in plant responses against pathogen attack [23–25]. Moreover, the CBL-CIPK modules are primarily implicated in modulating the level of ROS to mediate pathogen resistance. For instance, in tomato (*Solanum lycopersicum*), the SlCBL10-SlCIPK6 signaling pathway is involved in broad-spectrum antibacterial activity [24]. The SlCBL10-SlCIPK6 module is indispensable for the programmed cell death (PCD) triggered by pathogenic effectors. Additionally, overexpression of CIPK6 induces ROS accumulation by activating RBOHB in the plasma membrane.

To date, the understanding of the molecular mechanisms underlying BraCRa against *P. brassicae* is still limited. Notably, *P. brassicae* exists in diverse pathotypes, and the resistance of BraCRa to different pathotypes is also unclear [26]. In this study, we investigated the tissue-specific and subcellular localization of BraCRa, and verified the broad spectrum of *BraCRa* against *P. brassicae*. In addition, mRNA-seq was employed to explore the putative downstream components and pathways involved in BraCRa-mediated immune response in *B. rapa*. Finally, a Ca^2+^ sensor BraCBL1.2 was identified to be involved in the *BraCRa*-mediated clubroot resistance in Chinese cabbage.

## Results

### *BraCRa* confers Chinese cabbage race-specific resistance against *P. brassicae*

**Figure 1:**
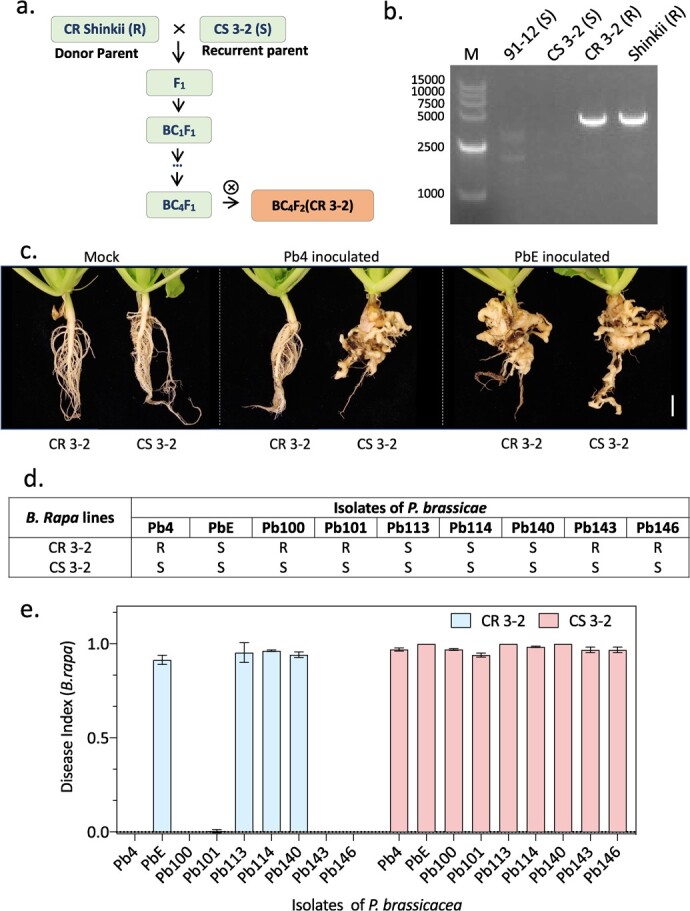
*BraCRa* confers *Brassica rapa* resistance against *Plasmodiophora brassicae*. **a** Development of CR 3–2 and CS 3–2 NILs. CR Shinkii (R) acted as the donor parent, and a clubroot susceptible Chinese cabbage line was designated as CS 3–2. **b** PCR verification of the present of *BraCRa* in the genome of CR 3–2. M is short for marker, and 91–12 is a *P. brassicae* susceptible cultivar. **c** Clubroot phenotype of CS 3–2 and CR 3–2 treated with sterile water (Mock), Pb4, or PbE at 42 dpi. Roots of 21 days old plants of inbred lines CR 3–2 and CS 3–2 were inoculated by sterile water (Mock), Pb4, or PbE pathotypes of *P. brassicae*. Gall formation was examined and photographed at 42 dpi in the roots of Chinese cabbage as sign of clubroot disease. Scale bar = 1 cm. **d***P. brassicae* resistance evaluation of Chinese cabbage CR 3–2 and CS 3–2 infected by nine different *P. brassicae* isolates (Pb4, PbE, Pb100, Pb101, Pb113, Pb114, Pb140, Pb143, and Pb146). Gall formation was examined at 42 dpi in the roots of Chinese cabbage as sign of clubroot disease. S refers to ‘susceptible to *P. brassicae*’, while R stands for ‘resistant to *P. brassicae*’. **e** Clubroot disease index of Chinese cabbage CR 3–2 and CS 3–2 infected by nine different *P. brassicae* isolates. The value was calculated at 42 dpi.

To assess the pathogenicity of *P. brassicae* and its ability to induce clubroot disease in the Chinese cabbage inbred line CR 3–2, we conducted an inoculation experiment using nine different Pbs on the roots of CR 3–2 and its near-isogenic line CS 3–2 as a susceptible control (Fig. 1a). According to Sinitic Clubroot Differential (SCD) set classification system [27], Pb4 belongs to pathotype 7, and Pb100, Pb101, Pb143, and Pb146 are pathotype 1. PbE is pathotype 16, and pb113, pb 114, and pb140 are pathotype 4. In addition, the presence of BraCRa in the genome of CR3–2 was verified by PCR (Fig. 1b). Disease resistance was evaluated at 42 days post inoculation (dpi). The susceptible control CS 3–2 exhibited disease symptoms to all Pbs (Fig. 1d). In contrast, the clubroot-resistant line CR 3–2 displayed resistance to Pb4, Pb100, Pb101, Pb143, and Pb146, while showing susceptibility to PbE, Pb113, Pb114, and Pb140 (Fig. 1d). A further phenotypic analysis of gall formation indicated that comparable galls were observed on the roots of CS 3–2 upon inoculation of Pb4 and PbE (Fig. 1c). The disease index was calculated according to a formula (Fig. 1e; Fig. S1, see online supplementary material). Inoculation with PbE resulted in similar gall formation on the roots of both CR 3–2 and CS 3–2, while Pb4 was only able to induce gall formation on the roots of CS 3–2 (Fig. 1c). Based on these results, Pb4 and PbE were chosen for further transcriptomic analysis of CR 3–2.

### *BraCRa* expressed in roots of cruciferous plants and induced by Pb4 treatment

**Figure 2:**
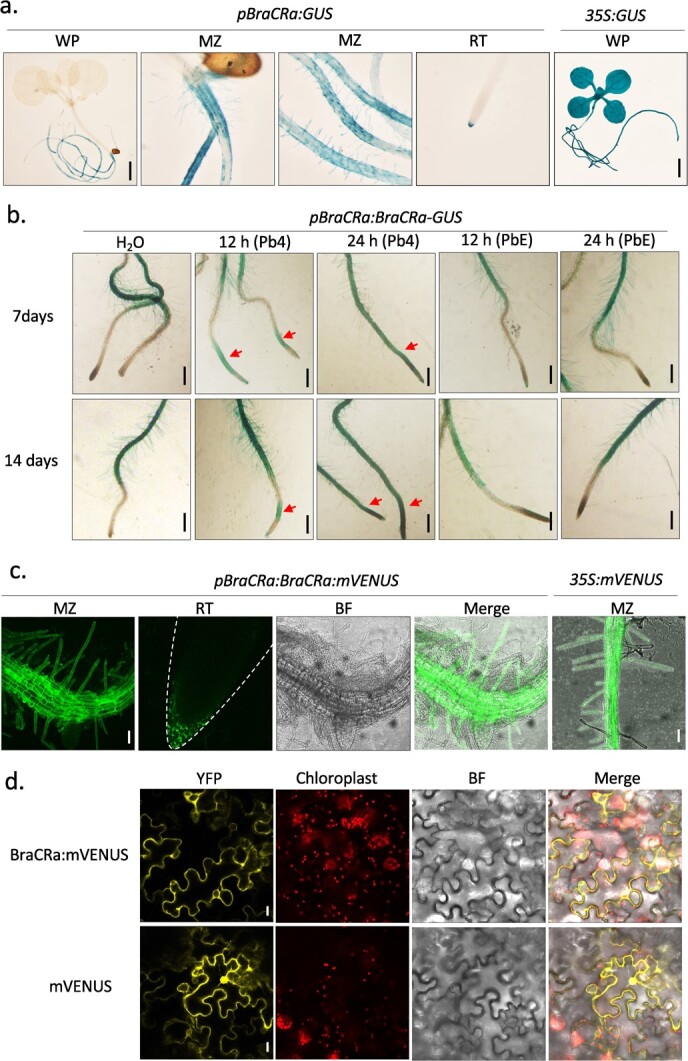
Tissue-specific expression pattern of *BraCRa* in *Arabidopsis* and subcellular localization of BraCRa in *Nicotiana benthamiana*. **a** Histochemical staining of 7-day-old *Arabidopsis* Col-0 seedlings transformed with *pBraCRa:GUS* or *35S:GUS* (as positive control). MZ, maturation zone of root; RT, root tip; WP, whole plant; scale bar = 2.5 mm. **b** Histochemical staining of 7 days and 14 days old Col-0 *Arabidopsis* seedlings transformed with *pBraCRa:BraCRa-GUS* treated with H_2_O (negative control), Pb4 and PbE for 12 and 24 h. Red arrows indicate the stained elongation zone; scale bar = 100 μm. **c** Confocal images of yellow fluorescence of 14-days-old Col-0 Arabidopsis seedlings transformed with *pBraCRa:BraCRa:mVENUS* and *35S:mVENUS* (positive control). BF, bright field; MZ, maturation zone; RT, root tip; scale bar = 0.5 mm. Dash line outlines the root tip. **d** Confocal images of yellow fluorescence of 4-weeks-old tobacco leaves infiltrated with *35S:BraCRa:mVENUS* or *35S:mVENUS* (positive control). YFP, yellow florescence protein; bright field; scale bar = 25 μm.

Because *P. brassicae* is a soil-borne pathogen that typically infects plants through the root hairs and cortical layers of the roots, causing disease in the plant [1], we sought to investigate the spatial and temporal expression patterns of resistance gene *BraCRa* in the root tissue of cruciferous plants. We constructed a binary vector containing the *GUS* reporter gene linked to the *BraCRa* gene promoter (2000 bp upstream of the start codon) and stably transformed it into the wild-type *Arabidopsis* Col-0 genome. The expression pattern of the *BraCRa* resistance gene in *Arabidopsis* was evaluated using GUS histochemical staining. As shown in Fig. 2a, the GUS gene driven by the *BraCRa* gene promoter was not expressed in the aboveground parts, such as the hypocotyl, leave, and petioles, of 7-day-old *Arabidopsis* seedlings. Instead, GUS staining was observed in the maturation zones of the main and lateral roots, root hair cells, and root caps (Fig. 2a). Moreover, in the transgenic *Arabidopsis* line expressing *pBraCRa:BraCRa-mVENUS*, fluorescence was also localized in the root hair cells and root cap, rather than in the meristematic zone and elongation zone (Fig. 2c). Therefore, we concluded that the *BraCRa* resistance gene was mainly expressed in the roots of *Arabidopsis*, especially in the maturation zones of the main and lateral roots, and was primarily located in cortical cells and root hair cells. To investigate whether treatment of Pb4 and PbE can promote the expression of *BraCRa* in plant roots, we performed GUS staining of 7 days and 14 days old seedlings treated with water as control, and with Pb4 and PbE for 12 and 24 hours. The results showed that Pb4, but not PbE treatment can induce the expression of *BraCRa* in elongation zone after 12 hours treatment, and in whole roots after 24 hours treatment. To investigate the subcellular localization of BraCRa, we constructed *p35S:BraCRa-mVENUS* in a binary vector. Through transient protein expression in tobacco leaves by *Agrobacterium*-mediated infection, the fluorescence signal was mainly observed at the plasma membrane and in the cytoplasm of the epidermal cells of tobacco leaves using confocal microscopy (Fig. 2d), while the fluorescence of the positive control was dispersed across the whole plant cell (Fig. 2d). Thus, we concluded that the BraCRa protein was mainly localized at the plasma membrane and in the cytoplasm of plant cells rather than in the nucleus.

### Data mining of RNA-seq and analysis of DEGs

In this study, we examined the expression levels of mRNA in the roots of CR 3–2 through RNA-seq, after inoculating with two Pbs, PbE, and Pb4. Transcriptome sequencing (RNA-seq) of Chinese cabbage CR 3–2 roots yielded 15 cDNA libraries, which were classified into five groups: CK-0d, PbE-8d, Pb4-8d, PbE-23d, and Pb4-23d. We obtained 7–10 × 10^7^ clean reads with a GC content ranging from 45.88 to 49.32% and Q30 ratios exceeding 98%. The proportion of reads aligned to the reference genome ranged from 58.73 to 77.91%, with most of the reads aligning to a single location, indicating that the sequencing results were highly reliable and suitable for further analysis. Finally, samples of CK-0d, PbE-8d, Pb4-8d, PbE23d, and Pb423d yielded 325, 421, 323, 325, and 686 specific transcripts, respectively (Fig. 3a). Thereafter, we analysed differentially expressed genes (DEGs) in response to *P. brassicae* (Fig. 3b). Specifically, there were 1830, 5530, 1097, and 11 843 DEGs in the pb4-8d, pb4-23d, pbE-8d, and pbE-23d samples, respectively, when compared to the CK-0d sample (Fig. 3b; Table S1, see online supplementary material). Notably, 10 708 DEGs were identified in the comparison of PbE-8d-vs-PbE-23d, and 4985 DEGs were identified in the Pb4-8d-vs-Pb4-23d comparison (Fig. 3b; Table S1, see online supplementary material). Additionally, there were only 55 upregulated DEGs and 74 downregulated DEGs in the comparison of Pb4-8d-vs-pbE-8d, whereas there were 830 DEGs in the comparison of Pb4-23d-vs-PbE-23d (Fig. 3b; Table S1, see online supplementary material). These results indicate that 23 dpi is the key time point for screening out crucial DEGs downstream of *BraCRa*, as the gene expression levels of CR 3–2 treated with PbE and Pb4 were significantly different at 23 dpi.

**Figure 3:**
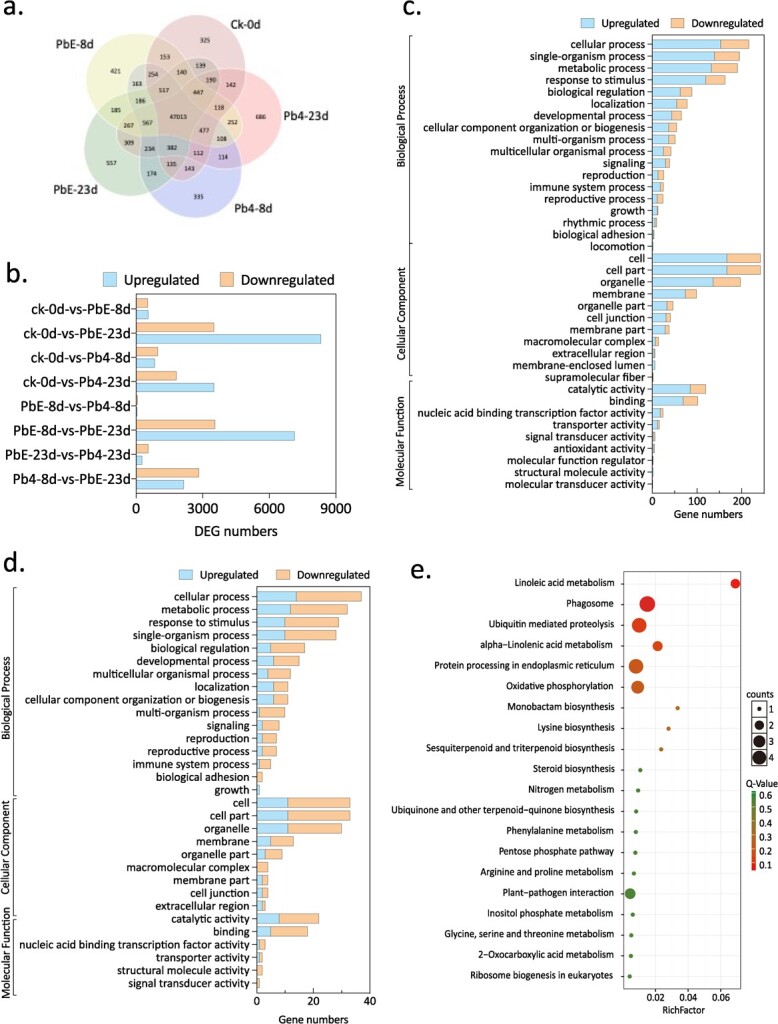
DEG analysis of RNA-seq data. Number of differentially expressed transcripts in different samples (**a**). Number of up- and downregulated DEGs in different comparisons of paired samples (**b**). GO terms of DEGs in response to infection by PbE and Pb4 at 8 and 23 dpi, (**c**) pbE-8d-vs-pb4-8d, (**d**) pbE-23d-vs-pb4-23d. Top 20 KEGG enrichment pathways of DEGs in CR 3–2 upon infection with PbE and Pb4 at 8 and 23 dpi, (**e**) pbE-8d-vs-pb4-8d, (**f**) pbE-23d-vs-pb4-23d. **g** Heatmap of 34 DEGs enriched in immune system process and response to stimuli (GO analysis), and plant–pathogen interaction (KEGG analysis) at 8 dpi (PbE-8d-vs-Pb4-8d) and 23 dpi (PbE-23d-vs-Pb4-23d). Red rectangles mark the four Ca^2+^-related components: *TCONS 00005389* (*BraCML11*), *Bra025696* (*BraCPK10*), *Bra012655* (*BraCBL1*.*2*), and *TCONS 00072129* (*BraCNGC20*).

**Figure 3:**
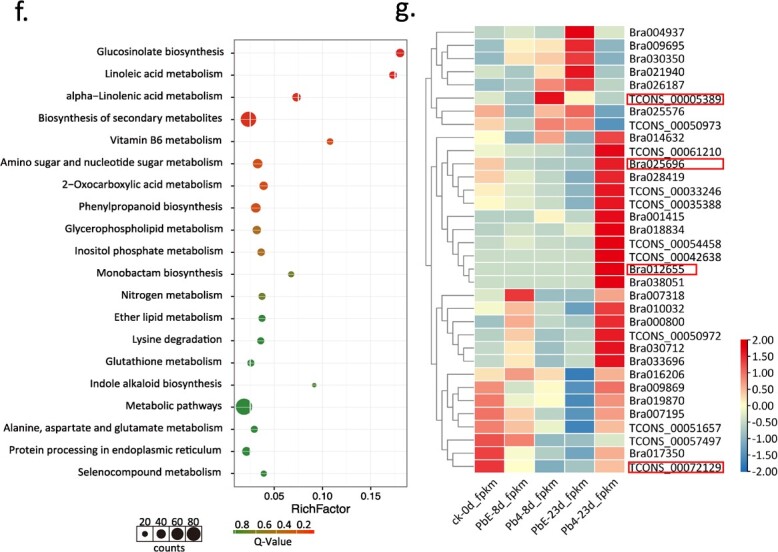
Continued.

In living organisms, transcript variants collaborate to execute various biological functions. The identification of significantly enriched Gene Ontology (GO) or Kyoto Encyclopedia of Genes and Genomes (KEGG) pathways among differentially expressed transcripts can enhance our understanding of their biological functions. To accomplish this, we conducted GO classification (Fig. 3c and d) and KEGG annotation (Fig. 3e and f). The GO classification results revealed that the DEGs in the Pb4-8d-vs-pbE-8d and Pb4-23d-vs-pbE-23d groups were predominantly enriched in ‘catalytic activity’ and ‘binding’ in molecular function, ‘cellular process’, ‘single-organism process’, and ‘metabolic process’ in biological process, and ‘cell’, ‘cell part’, and ‘organelle’ in cellular component (Fig. 3c and d). The DEGs of Pb4-8d-vs-pbE-8d were significantly enriched in pathways such as ‘Linoleic acid metabolism’, ‘Phagosome’, ‘Ubiquitin-mediated proteolysis’, and ‘alpha-Linolenic acid metabolism’ based on KEGG annotation. Likewise, the DEGs of Pb4-23d-vs-pbE-23d exhibited significant enrichment in signaling pathways, including ‘Glucosinolate biosynthesis’, ‘Linoleic acid metabolism’, and ‘alpha-Linolenic acid metabolism’ based on KEGG annotation. To assess the accuracy of transcriptome sequencing, qRT-PCR was employed to confirm differential gene expression (Fig. S2, see online supplementary material). Nine DEGs (*Bra004057*, *Bra016466*, *Bra039948*, *TCONS_00093390*, *Bra016602*, *Bra017350*, *Bra030988*, *Bra033991*, and *TCONS_001539259*) associated with the linoleic acid and alpha-linolenic acid metabolism pathways were chosen. At 8 dpi, *Bra016602* and *Bra030988* were upregulated in the PbE-8d samples. At 23 dpi, *Bra004057*, *Bra016466*, *Bra039948*, and *TCONS_00093390* were upregulated, whereas *Bra016602*, *Bra017350*, *Bra030988*, *Bra033991*, and *TCONS_001539259* were downregulated in the PbE-23d samples (Fig. S2, see online supplementary material). Moreover, seven DEGs (*Bra013011*, *Bra0029355*, *Bra032734*, *TCONS_0029640*, *TCONS_00045498*, *TCONS_00045499*, and *TCONS_00075482*) associated with glucosinolate biosynthesis pathways were selected for validation. At 8 dpi, *Bra013011*, *Bra0029355*, *Bra032734*, *TCONS_0029640*, *TCONS_00045498*, *TCONS_00045499*, and *TCONS_00075482* were downregulated in the PbE-8d samples, while they were upregulated in the PbE-23d samples at 23 dpi (Fig. S2, see online supplementary material). Collectively, RNA-seq has yielded high quality data set for further analysis.

### Ca^2+^ sensor BraCBL1.2 is implicated in BraCRa-mediated clubroot resistance

**Figure 4:**
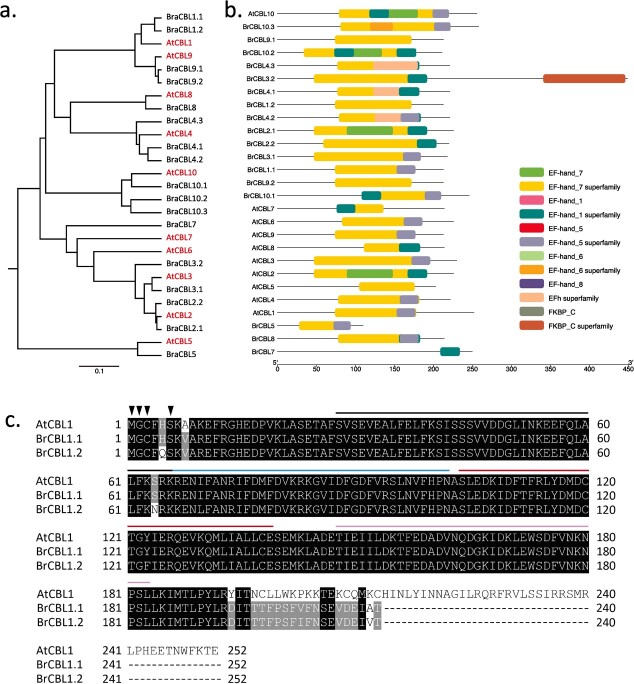
BraCBL1.2 acts as a Ca^2+^ sensor functioning downstream of the BraCRa-mediated immune response in *Brassica rapa*. **a** Phylogenic tree of homologous genes of BraCBL1.2 in *Arabidopsis* and *B. rapa*. Names in red mark the genes of *Arabidopsis*, while names in black mark the genes of *B. rapa*. **b** Protein domain architecture of the homologous gene of BraCBL1.2 in *Arabidopsis* and *B. rapa*. The axis below shows the length of the proteins in amino acid numbers. The conserved domains of proteins were evaluated using the NCBI conserved domains database (CDD) and visualized via Tbtools [30]. EF-hand domains confer proteins with the Ca^2+^-binding ability, and FKBPs are short for FK506-binding proteins. **c** Primary protein sequence alignment of AtCBL1, BraCBL1.1, and BraCBL1.2. The black background signifies the conserved amino acids, while the letters in black exhibit the different amino acids. The dashed line indicates that no amino acids are present. Lines in black, blue, red, and purple mark the four EF-hands of AtCBL1, and the inverted black triangle indicates the conserved amino acid sites that are subjected to lipid modification.

To identify potential downstream components of BraCRa, a selection of 34 DEGs enriched in the ‘immune system process’ and ‘response to stimuli’ of GO terms, as well as the ‘plant–pathogen interaction’ of the KEGG pathway, were compared (Fig. 3g). The heatmap analysis revealed that the majority of these 34 DEGs exhibited the highest expression level at 23 dpi, particularly under Pb4 infection. Four Ca^2+^-related genes, *TCONS 00072129* (*BraCNGC20*), *TCONS 00005389* (*BraCML11*), *Bra02569* (*BraCPK10*), and *Bra012655* (*BraCBL1*.*2*), were identified among these 34 DEGs. *TCONS 00072129*(*BraCNGC20*) exhibited the highest transcription level in the control group treated with sterile water (Ck-0d), while *TCONS 00005389* (*BraCML11*), *Bra02569* (*BraCPK10*), and *Bra012655* (*BraCBL1*.*2*) displayed the highest expression level at 8 or 23 dpi under Pb4 infection. These findings suggest that TCONS 00005389 (BraCML11), Bra02569 (BraCPK10), and Bra012655 (BraCBL1.2) may function as putative Ca^2+^ sensors involved in the BraCRa-mediated immune response. Considering the limited knowledge regarding CMLs in plant immune responses and the well-established role of CBLs in plant immune responses, Bra012655 (BraCBL1.2) was selected for further analysis. Sequence alignment of the coding sequence (CDS) revealed that *Bra012655* is a homologous gene of *AtCBL1* (*AT4G17615*) in *Arabidopsis* (Fig. 4a and c). AtCBL1, known as a calcium ion sensor, detects changes in the Ca^2+^ concentration and interacts with downstream CIPKs, thereby activating downstream proteins [28].

**Figure 5:**
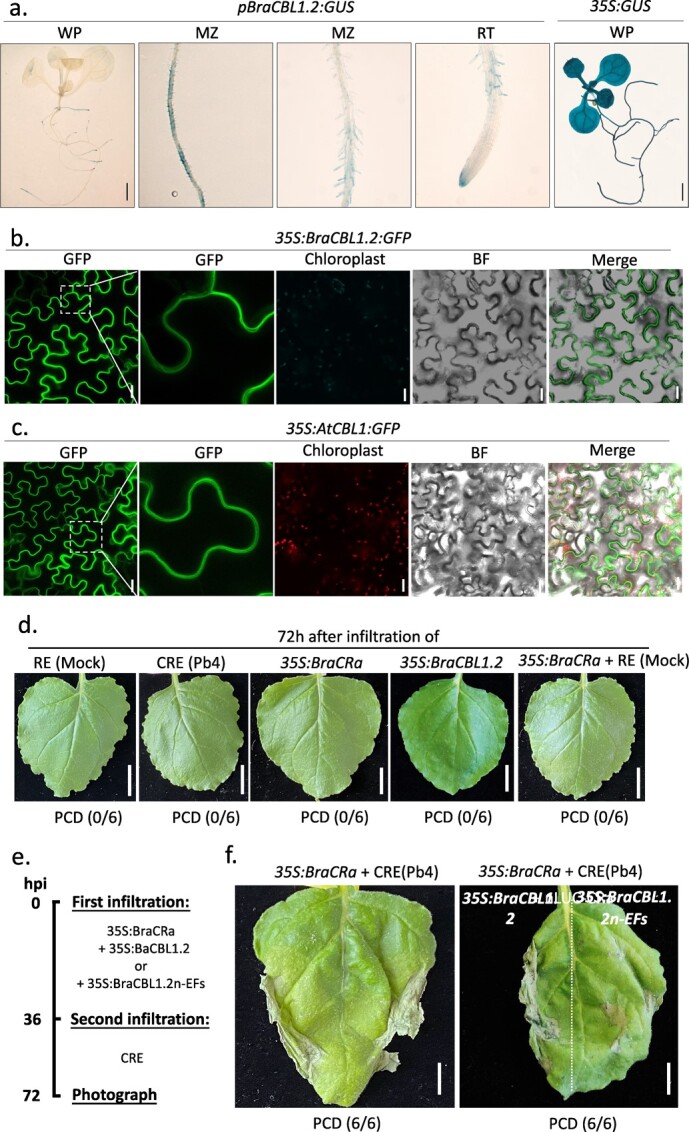
Tissue-specific expression pattern of *BraCBL1*.*2* in *Arabidopsis* and subcellular localization of BraCBL1.2 in *Nicotiana benthamiana*. **a** Histochemical staining of 7-day-old *Arabidopsis* Col-0 seedlings transformed with *pBraCBL1*.*2:GUS* or *p35S:GUS* (as a positive control). MZ, maturation zone of root; RT, root tip; WP, whole plant; scale bar = 2.5 mm. Confocal images of green fluorescence of 4-week-old tobacco leaves infiltrated with *35S:BraCBL1*.*2-GFP* (**b**) or *35S:AtCBL1-GFP* (**c**). GFP, green florescence protein; BF, bright field; scale bar = 25 μm. (**d**) 72 hours after infiltration of RE(Mock) and CRE, and transient expression of 35S:BraCRa, 35S:BraCBL1.2, and 35S:BraCRa+RE(Mock). Scale bar = 2 cm (**e**) Procedures of tobacco infiltration and plasmid combinations used for (f), and (**f**) shows PCD of tobacco leaves. Scale bar = 1 cm.

In total, 17 *CBL* genes were identified in *B. rapa*, including *BraCBL1*.*1* and *BraCBL1*.*2* [29]. Moreover, conserved domain analysis (Fig. 4b and c) revealed that both BraCBL1.1 and BraCBL1.2 contained four EF-hand domains and showed highly homologous amino acid sequences with AtCBL1. To reveal the evolutionary relationships between AtCBLs and BraCBLs, 27 CBL protein sequences were used to conduct a phylogenetic tree using Mega 6.0. The results showed a close evolutionary relationship between BraCBL1.1, BraCBL1.2, and AtCBL1 (Fig. 4a). Furthermore, the amino acid sequence alignment of AtCBL1, BraCBL1.1, and BraCBL1.2 demonstrated high conservation with the four EF-hand domains and N-terminal lipid-modified amino acids among these proteins (Fig. 5c). These findings suggest that BraCBL1.2 likely possesses Ca^2+^-binding ability and undergoes lipid modification for translocation to the plasma membrane.

**Figure 6:**
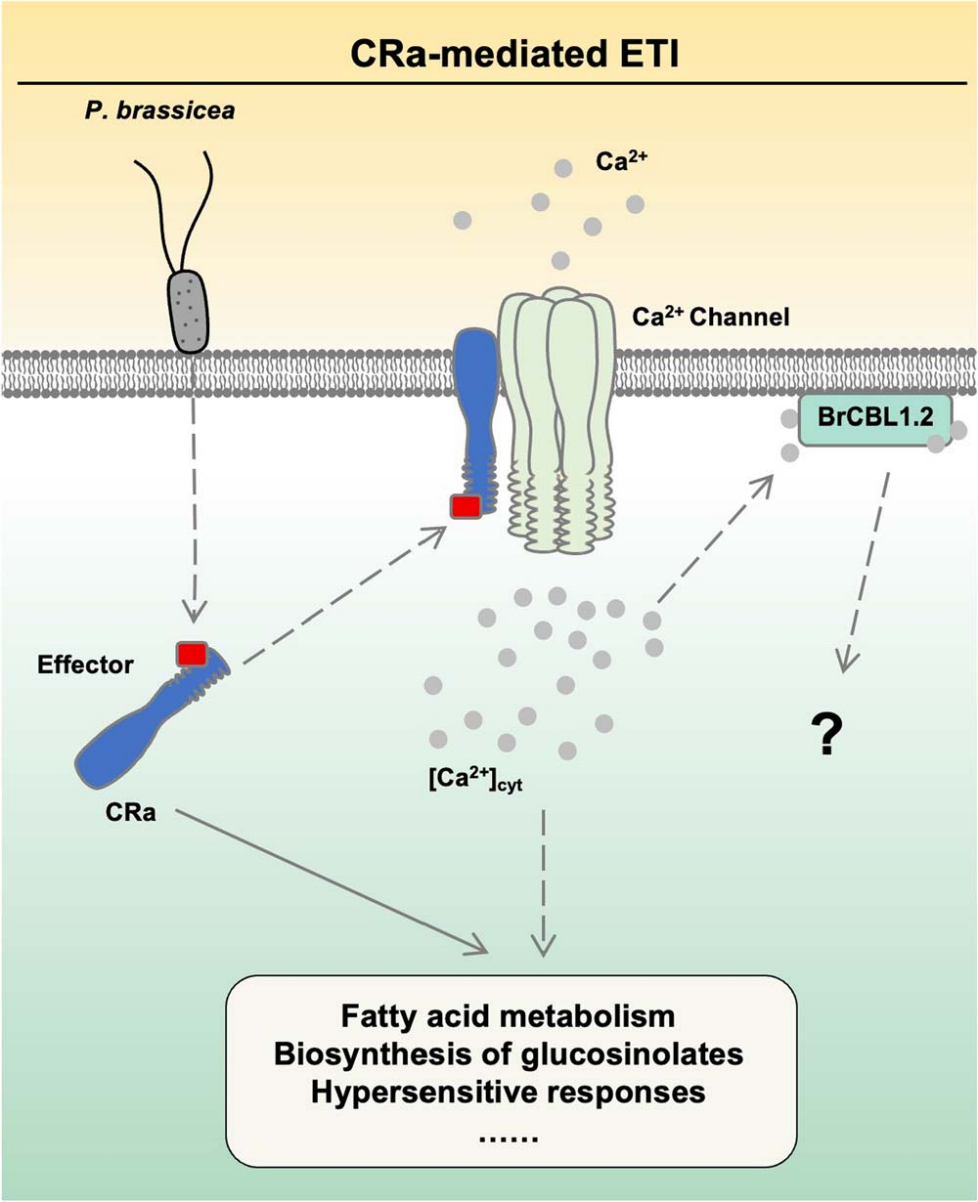
Schematic representation of BraCRa-mediated ETI response in root hair cells of *Brassica rapa* upon infection by *Plasmodiophora brassicae*. Upon *P. brassicae* infection, ETI responses are elicited in root hair cells to defend the invasion by *P. brassicae*. Once *P. brassicae* secretes the effector into the cytoplasm, a specific ETI response is initiated. BraCRa associates with the putative effectors and translocates to the PM, where BraCRa recruits putative RNL or unknown Ca^2+^ channels to trigger the cytosolic Ca^2+^ increases (shown as [Ca^2+^]_cyt_). Subsequently, the supposed cytosolic Ca^2+^ increases are sensed by BraCBL1.2 at the PM, which transmits the encoded immune signals to unknown downstream components. Meanwhile, the activated BraCRa can also modulate the fatty acid metabolism and biosynthesis of glucosinolates to mediate *P. brassicae* resistance. The dashed lines indicate the unproven pathways. Gray dots represent Ca^2+^. Proteins are depicted in different colors and forms.

### BraCBL1.2 shares similar spatial and temporal expression pattern with BraCRa

To ensure that BraCBL1.2 functions downstream of BraCRa in plant root hair cells, it was crucial to assess the spatial and temporal expression patterns of BraCBL1.2. A binary vector containing the GUS reporter gene driven by the BraCBL1.2 gene promoter (1000 bp upstream of the start codon) was constructed and stably transformed into the wild-type *Arabidopsis* Col-0 genome. As depicted in Fig. 5a, GUS expression driven by the BraCBL1.2 promoter was predominantly detected in the root region of 7-day-old *Arabidopsis* seedlings, particularly in the maturation zone and root hair cells. This observation indicates that BraCBL1.2 is primarily expressed in the maturation zone of *Arabidopsis* roots and is mainly located in cortical cells, including root hair cells. These results suggest that BraCBL1.2 shares a similar spatial and temporal expression pattern to BraCRa.

To investigate the subcellular localization of BraCBL1.2, BraCBL1.2 was fused with green fluorescent protein (GFP) using a binary vector. Transient protein expression was carried out in tobacco leaves, and the resulting fluorescence signals were observed using confocal microscopy three days after *Agrobacterium* infiltration. As depicted in Fig. 5b and c, both BraCBL1.2-GFP and AtCBL1-GFP were found to localize at the plasma membrane of tobacco leaf epidermal cells, which aligns with the conclusions drawn from previous studies [31]. Furthermore, the interaction between BraCRa and BraCBL1.2 was investigated in yeast two-hybrid and luciferase complementation assays (Fig. S4a and b, see online supplementary material). However, no direct interaction was observed between BraCRa and BraCBL1.2. To verify the involvement of BraCBL1.2 in BraCRa-mediated clubroot resistance, we established the tobacco-based HR assay. In this assay, the infiltration effects of the clubroot extract (CRE) of Pb4, healthy root extract (RE), and agrobacteria-mediated *35S:BraCRa*, *35S:BraCBL1.2*, and *35S:BraCRa* transformation were examined in tobacco leaves. None of them induced PCD in tobacco leaves 72 hours post infiltration (hpi) (Fig. 5d). Intriguingly, when the CRE was infiltrated in *35S:BraCRa-*expressing tobacco leaves, severe necrosis were observed (Fig. 5f). Therefore, this tobacco-based HR assay suggests that BraCRa successfully induces HR in tobacco leaves. To investigate the involvement of BraCBL1.2 in BraCRa-mediated the ETI immune response, we co-transformed *35S:BraCRa* and *35S:BraCBL1.2* in tobacco leaves, and *35S:BraCRa* and *35S:BraCBL1.2-EFs* in tobacco leaves. *BraCBL1.2n-EFs* is a truncated version of *BraCBL1.2*, and has only the N-terminal conserved PM-localized domain and four EF-hands domains (Fig. S5, see online supplementary material). The tobacco-based HR assay showed that *BarCBL1.2n-EFs* dramatically inhibited the PCD of tobacco leaves, in addition, *BraCBL1.2* promoted the PCD of tobacco leaves (Fig. 5f). In addition, we constructed one *Arabidopsis* transgenic line *#7* expressing *pBraCRa:BraCRa*, and the *BraCBL1.2* overexpression line in *#7* background (Fig. S6, see online supplementary material). The clubroot phenotype analysis showed that *#7* exhibited resistance against Pb4, and overexpression of *BraCBL1.2* in #7 enhanced its resistance against *P. brassicae* (Fig. S6, see online supplementary material). Collectively, these findings suggest that BraCBL1.2 functions as a Ca^2+^ sensor localized in the plasma membrane of root hair downstream of BraCRa, playing a role in decoding Ca^2+^ signaling to mediate ETI response.

## Discussion

As a soil-borne protist pathogen, the life cycle of *P. brassicae* is mainly comprised of three stages: resting spore stage, primary infection stage, and secondary infection stage. The primary and secondary infection stages involve symbiosis with plants [3]. Typically, the residual root galls of diseased plants remain in the soil and release zoospores, thereby becoming new sources of contamination after decay [32]. A previous study validated that root hair infection was observed in both *P. brassicae*-resistant and -susceptible hosts, but no infection was observed during the cortical infection stage in resistant roots [33]. In this study, we found that the *BraCRa* gene was primarily expressed in the mature zone of *Arabidopsis* roots, specifically in root hair cells. These findings suggest that BraCRa plays a key role during the primary infection stage in conferring resistance to *P. brassicae* in Brassicaceae plants. Moreover, BraCRa was predominantly localized in the cytoplasm and cell membrane (Fig. 2). Upon encountering a host plant, motile spores can infect plant root hairs and deliver virulent proteins into the cytoplasm of root hair cells, where they are perceived by BraCRa to trigger unknown downstream immune responses. Therefore, BraCRa successfully prevents the infection of *P. brassicae* and the development of primary plasmodia and zoosporangia in the roots of *B. rapa*.

The primary objective of this study was to investigate the downstream regulation pathways and key gene members involved in downstream BraCRa-mediated clubroot resistance in Chinese cabbage using transcriptomic analysis. Phenotypic analysis was conducted using Chinese cabbage cultivar CS 3–2 and its near-isogenic line CR 3–2 to screen for the desired physiological races of *P. brassicae*. CR 3–2 exhibited resistance to the multiple races of *P. brassicae*, suggesting the presence of shared effectors among these races. Specifically, CR 3–2 showed resistance to Pb4 but not to PbE, indicating the potential synthesis and secretion of different virulent proteins by Pb4 and PbE as effectors delivered into root hair cells.

The KEGG annotation of DEGs of CR 3–2 in response to Pb4 and PbE treatments revealed that the significant DEGs were mainly enriched in the fatty acid metabolism pathway, including linoleic acid metabolism, α-linolenic acid metabolism, and the glucosinolate biosynthesis pathway (Fig. 3; Fig. S3, see online supplementary material). Typically, fatty acid metabolism plays a crucial role in the host–pathogen interaction [34, 35]. However, the DEGs related to linoleic acid and α-linolenic acid metabolism were downregulated upon *P. brassicae* infection, indicating their dispensability for *P. brassicae* resistance. In contrast, DEGs related to the biosynthesis of methionine-derived aliphatic glucosinolates were highly induced at 8 dpi but not at 23 dpi. These findings indicate that aliphatic glucosinolates are induced upon *P. brassicae* infection, which aligns with a previous study demonstrating lower levels of single and total aliphatic and indolic glucosinolates in susceptible cultivars compared to resistant cultivars [36]. Unlike indole glucosinolates, aliphatic glucosinolates are involved in defense mechanisms by releasing toxic thiocyanates and isothiocyanates, while indole glucosinolates are implicated in auxin biosynthesis. Elevated auxin levels are associated with large root galls, indicating that indole glucosinolates may directly or indirectly contribute to the extent of disease development [37]. Additionally, susceptible roots exhibited increased accumulation of aliphatic, indolic, and aromatic glucosinolates in *Brassica napus*, whereas only aromatic glucosinolates were significantly increased in *Matthiola incana* [33]. Furthermore, this study revealed a negative correlation between the glucosinolate biosynthesis pathway and the linoleic acid and alpha-linolenic acid pathways during *P. brassicae* infection, which contradicts previous research showing that both resistant and susceptible plants exhibit enhanced levels of jasmonic acid, particularly in susceptible roots [33]. Taken together, *B. rapa* may differ in glucosinolate composition and JA content upon *P. brassicae* infection compared to *M. incana* and *B. napus*.

Recent research has demonstrated that many plant ETI immune-related R proteins act as calcium-permeable channels, highlighting the importance of identifying calcium receptors downstream of ETI immune responses [19]. In this study, transcriptome analysis of the Chinese cabbage line CR 3–2 identified a significant number of DEGs, including the *Bra012655* gene (*BraCBL1*.*2*). Phylogenetic analysis of homologous genes in the CBL gene family of cabbage and *Arabidopsis*, along with sequence alignment, indicated that BraCBL1.2 shares conserved EF-hand domains and N-terminal amino acids responsible for plasma membrane localization. This led us to infer that BraCBL1.2 in *B. rapa* may also participate in decoding calcium signals in the ETI immune response under the regulation of BraCRa. Subsequent experiments investigating the tissue-specific expression and subcellular localization of BraCBL1.2 showed its expression in the root hair cells of *Arabidopsis* localized at the plasma membrane, which corresponds to the putative function site of BraCRa. BraCBL1.2 and BraCRa exhibited similar spatiotemporal expression patterns in *Arabidopsis*, suggesting that BraCBL1.2 may decode the BraCRa-mediated ETI calcium signal (Fig. 6). Additionally, we developed a tobacco-based HR assay to evaluate the involvement of BraCBL1.2 in BraCRa-mediated clubroot resistance. In this assay, BraCBL1.2 promotes the PCD of tobacco leaves. However, a truncated BraCBL1.2, BraCBL1.2n-EFs inhibits the PCD of tobacco leaves. This could be ascribed to that BraCBL1.2n-EFs perceives the Ca^2+^, but could not delay signal to downstream components, thereby inhibiting the BraCRa-mediated ETI response. Therefore, subsequent work will focus on investigating potential components downstream of BraCRa in *B. rapa*, starting with BraCBL1.2.

## Materials and methods

### Plant materials and growth conditions

Two Chinese cabbage NILs, clubroot-resistant line CR 3–2 and clubroot-susceptible line CS 3–2, were used in this study. CR 3–2 carries the clubroot resistance gene *BraCRa*, which confers resistance to Pb4 but not to PbE. The Chinese cabbage seeds sown in soil were grown in 32-well multi-pots and placed in a growth chamber at 25/18°C with a 16/8 h light/dark cycle. One *Arabidopsis* ecotypes, Col-0 were cultivated in a growth chamber at 23/18°C with a 16/8 h light/dark cycle. *Nicotinana benthamiana* was cultivated in a growth chamber at 23/18°C with a 12/12 h light/dark cycle. *Arabidopsis* transformation was performed with *Agrobacterium* (strain GV3101) using the floral dip method.

### *P. brassicae* inoculation and tissue sampling

Nine Pbs were used in this study, and the pathotype classification was evaluated according to Pang *et al.* [27]. For inoculation, the roots of 21-day-old CS 3–2 and CR 3–2 seedlings were inoculated with a suspension of *P. brassicae*, while ck was treated with equal amounts of sterile water. The roots of CR 3–2, which were inoculated with Pb4 or PbE, were collected at 8 and 23 dpi, while the roots of the control were collected at 0 dpi. Freshly cleaned roots were frozen immediately in liquid nitrogen and stored at −80°C. Each sample included the roots of 10 plants that were blended together, and three biological replicates were included. Finally, 15 samples of CR 3–2 were collected for high-throughput RNA-seq and qRT-PCR analysis.

### RNA preparation, cDNA library construction, and sequencing

The total RNA of each sample was extracted using TRIzol reagent (Invitrogen, Carlsbad, CA, USA) following the manufacturer’s instructions. Subsequently, DNase I (Takara Bio, Dalian, China) was applied to the RNA samples to digest the residual genomic DNA. The concentration and purity of RNA were assessed using a NanoDrop-2000 spectrophotometer (Thermo Fisher Scientific, Wilmington, DE, USA). High-quality RNA samples were selected for the cDNA library and small RNA library on the basis of OD A_260_/A_280_ (1.9–2.1), A_260_/A_230_ (>2.0), and RIN ≥ 9.0. Moreover, an RNA Nano 6000 Assay Kit was used to assess the integrity of RNA with the Agilent Bioanalyzer 2100 system, and the Epicentre Ribo Zero rRNA Kit (Illumina, San Diego, USA) was utilized to remove rRNA.

RNA-seq was subsequently performed by Gene Denove Biotechnology Co. (Guangzhou, China) using Illumina HiseqTM 2500 (Illumina, San Diego, USA). First, a strand-specific library was constructed. After removing rRNA from the extracted total RNA, most of the coding RNA and ncRNA were reserved. The obtained RNA was randomly broken into short fragments, which acted as a template to synthesize the first-strand cDNA with random primers. Subsequently, the second-strand cDNA was synthesized, followed by purification (QIAquick PCR kit; Guangzhou, China), end repair, poly(A) addition, ligation of Illumina sequencing adapters, and degradation by the UNG (Uracil-N-Glycosylase; QIAGEN, Guangzhou, China). Furthermore, the fragments obtained from the previous steps were further selected and filtered using agarose gel electrophoresis to proceed with PCR amplification. Finally, the mRNA library was sequenced using Illumina HiSeqTM 4000 (Illumina, San Diego, USA) by the Gene Denove Biotechnology Co. (Guangzhou, China).

### Transcriptomic data analysis

Before analysing the RNA-seq data, low-quality reads, including those containing adapters, having either more than 10% of unknown nucleotides (N) or more than 50% of low quality (Q-value ≤20) bases, and consisting of all A bases, were filtered out from the raw data. First, the remaining clean reads were mapped to the ribosomal RNA (rRNA) database using the short reads alignment tool Bowtie2 to remove matched reads accordingly. The remaining reads of each sample were further aligned to the *B. rapa* genome (Version 1.5, http://brassicadb.org/brad/) using TopHat2 software (version 2.1.1) to screen out reads with mismatch≤2. The low-quality reads that did not meet the screening criteria were further analysed to search for potential splice sites. In contrast, high-quality reads were used to reconstruct transcripts using Cufflinks software. All of the reassembled fragments were aligned to the *B. rapa* genome (Version 1.5, http://brassicadb.org/brad/).

### Differential expression analysis of mRNAs

The expression levels of transcripts were calculated and normalized by reads per kilo base per million mapped reads (RPKM, RPKM = exon mapped reads × 10^9^/ (total mapped reads × exon length). The edgeR software was used to identify differentially expressed transcripts, and the significant differentially expressed coding RNAs were determined by comparison between samples with fold change ≥2 and false discovery rate (FDR) <0.05. All DEGs were mapped to GO terms using the GO database (http://www.geneontology.org/) and enriched in the KEGG pathways. Finally, function classification and enrichment analysis were performed with Web Gene Ontology (WEGO) and topGO software, respectively.

### qRT-PCR analysis of DEGs and target genes

qRT-PCR analysis was performed to validate the results of the transcriptomic analysis. First, RNA isolation was performed, as described above. The first-strand cDNA was then reverse-transcribed from RNA using the FastQuant RT Super Mix kit (KR108). The cDNA was mixed with SuperReal PreMix Plus reagent (Tiangen, Beijing) and primers, which were designed using Primer5.0 software and are listed in Table S2 (see online supplementary material). The Bio-Rad CFX 96 real-time PCR system was used to perform the qRT-PCR reactions with the following settings: 95°C for 15 min, followed by 40 cycles of 95°C for 10 s, 60°C for 20 s, 72°C for 25 s, and 72°C for 30 s. *Actin* and *18sRNA* were used as internal controls for mRNA. The specificity of amplification was evaluated by melting curve analysis. The relative expression of the target genes was calculated using the 2^-ΔΔCT^ method. All samples were evaluated individually with three biological and technical replicates.

### Histochemical GUS staining

For spatiotemporal expression analysis, the 2000 bp promoter regions of BraCRa and *BraCBL1*.*2* were amplified from the genomic DNA of Chinese cabbage and constructed into the binary vector pCAMBIA1300 to lead to the expression of the *uidA* reporter gene. For histochemical staining analysis, 7-day-old seedlings of wild-type *Col-0* transformed with *p35S:GUS* (positive control) and transgenic *Arabidopsis* lines carrying *pBraCBL1*.*2-uidA* were immersed in 2 mM 5-bromo-4-chloro-3-indolyl-β-D-glucuronide solution for 12 h. Subsequently, the stained seedlings were treated with 70% ethanol to remove chlorophyll. For Pb4 and PbE treatment, roots of seedlings were immersed into the suspension of *P. brassicae* for 12 h or 24 h.

### Transient protein expression in leaf epidermal cells of *N*. *benthamiana*

Transient protein expression in tobacco leaves was used to explore the subcellular localization of BraCRa and BraCBL1.2. For this purpose, *BraCBL1*.*2* was fused with *GFP* to construct the pCAMBIA1300*-*35S*-BraCBL1*.*2-GFP* plasmid, and the *BraCRa* gene was fused with mVENUS to construct pCAMBIA1300-35S*-BraCRa-mVenus*. First, the constructed vectors were transformed with *Agrobacterium GV3101:pmp90*. Leaves of 4-week-old tobacco were infiltrated by the transformed *Agrobacterium* with an OD600 value of 0.5. After cultivation under continuous weak lights for 3 days, the fluorescence signal of BraCRa:mVenus or BraCBL1.2-GFP was detected using confocal microscopy (Leica, DMI6000B) equipped with a Leica TCS SP5 laser scanning device. GFP was excited at 488 nm (Ar laser), and fluorescence was recorded at 500–525 nm. All images were photographed using a HCX PL APO lambada blue 63x/1.2 water-immersion objective and Leica confocal system software. pCAMBIA1300*-35S:mVENUS* and pCAMBIA1300*-35S:AtCBL1:GFP* were used as controls.

### Yeast two hybrid assay

The CDS of *BraCBL1*.*2* was constructed into the pGBKT7 vector to act as a bait protein, while *BraCRa* and *AtCIPK1* were constructed into pGADT7 to be prey. Both AD and BD vectors were co-transformed into the PJ69-4A yeast strain. Co-transformed yeast of different vector combinations was spotted on selective SD media lacking Leu and Trp (SD-LW) for the selection of positive double transformants. Selective media lacking Leu, Trp, and His (SD-LWH) supplemented with 0.5 mM 3-amino-1,2,4-triazole (3-AT) were used to select the interactions. All pictures were taken after 7 days of incubation at 30°C. A combination of AD-AtCIPK1 and BD-BraCBL1.2 acted as a positive control, while the combination of empty AD and BD vectors was a negative control.

### Luciferase complementation assay

Luciferase complementary assays were performed according to the published protocol [38]. The coding sequence of *BraCBL1*.*2* was constructed into pCAMBIA1300-cLUC, while *AtCIPK1* and *BraCRa* were fused to the carboxyl-terminal of nLUC in pCAMBIA1300. The empty pCAMBIA1300-cLUC and pCAMBIA1300-nLUC were taken as negative controls. Paired proteins were transiently expressed in *N*. *benthamiana* leaves via *Agrobacterium* GV3101. After 2–5 days of cultivation, tobacco was placed in the dark for around 6 min to quench fluorescence in the chloroplasts. Then, the leaves were removed and quickly smeared with 0.2 mM luciferin (3–4 drops from 1 ml syringe; dissolved in water; stocks stored in −20°C) using a finger pressed evenly on the reverse side of the leaves. Finally, pictures were taken with a CCD camera for 10–20 min (20 min recommended).

### Tobacco-based HR assay

For this assay, raw CRE and RE were extracted from roots of Pb4-infected and healthy cabbage roots by grind using mortar with water. Subsequently, the raw CRE and RE were centrifuged at 12000 *g* for 10 mins to harvest the clear supernatant, which was then filtered through 2.5 μm filter to get the final CRE and RE. The coding sequence of *BraCRa*, *BraCBL1.2n-EFs*, and *BraCBL1*.*2* were constructed into pCAMBIA1300-35S. Paired proteins were transiently expressed in *N*. *benthamiana* leaves via *Agrobacterium* GV3101. After 36 h cultivation, CRE were filtrated to the tobacco leaves. At last, the necrosis of tobacco leaves was documented by photograph.

### *Arabidopsis* transformation

We constructed *BraCRa* expression *Arabidopsis* line by transforming pCAMBIA1300-*pBraCRa-BraCRa-mVenus* plasmids into Col-0 through Agrobacterium-mediated transformation. To constitutively overexpress *BraCBL1.2* in Arabidopsis, the pCAMBIA1300*-*35S*-BraCBL1*.*2-GFP* plasmids were transformed into *pBraCRa:BraCRa #7* plants. To verify the disease resistance of wild type and transgenic *Arabidopsis* lines, 14-day-old seedlings were treated with of water or Pb4. The disease resistance was investigated on the 42 days after inoculation, and calculated desease index.

## Supplementary Material

Web_Material_uhad261

## Data Availability

The raw sequence data of RNA-seq reported in this study have been deposited in the National Center for Biotechnology Information (NCBI) Sequence Read Archive (SRA) data under accession number PRJNA999218. The *Arabidopsis thaliana* and *B. rapa* genome sequences are available in TAIR (http://www.arabidopsis.org) and BRAD (http://brassicadb.cn), respectively.
