# Supplementary material for: A Ca2+ sensor BraCBL1.2 involves in BraCRa-mediated clubroot resistance in Chinese cabbage
Source: Hortic Res. 2023 Dec 13;11(1):uhad261. doi: 10.1093/hr/uhad261 (PMC10828780; doi:10.1093/hr/uhad261)
Supplement: Web_Material_uhad261 [file web_material_uhad261.docx]

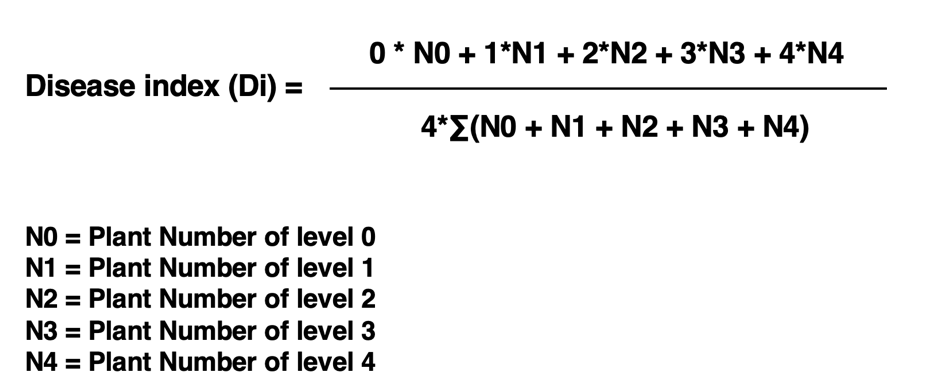


**Supplemental Figure 1. formula for calculating disease index.** Level 0 refers to healthy plants; Level 1 refers to the plants having small galls (diameter shorter than 0.5 cm) at side roots; Level 2 refers to the plants having big galls (diameter longer than 0.5 cm) at side roots, but no galls at main root; Level 3 refers to the plants having big galls (diameter longer than 0.5 cm) at main roots, but no big galls at side roots; Level 4 refers to the plants having big galls (diameter longer than 0.5 cm) at both main and side roots.


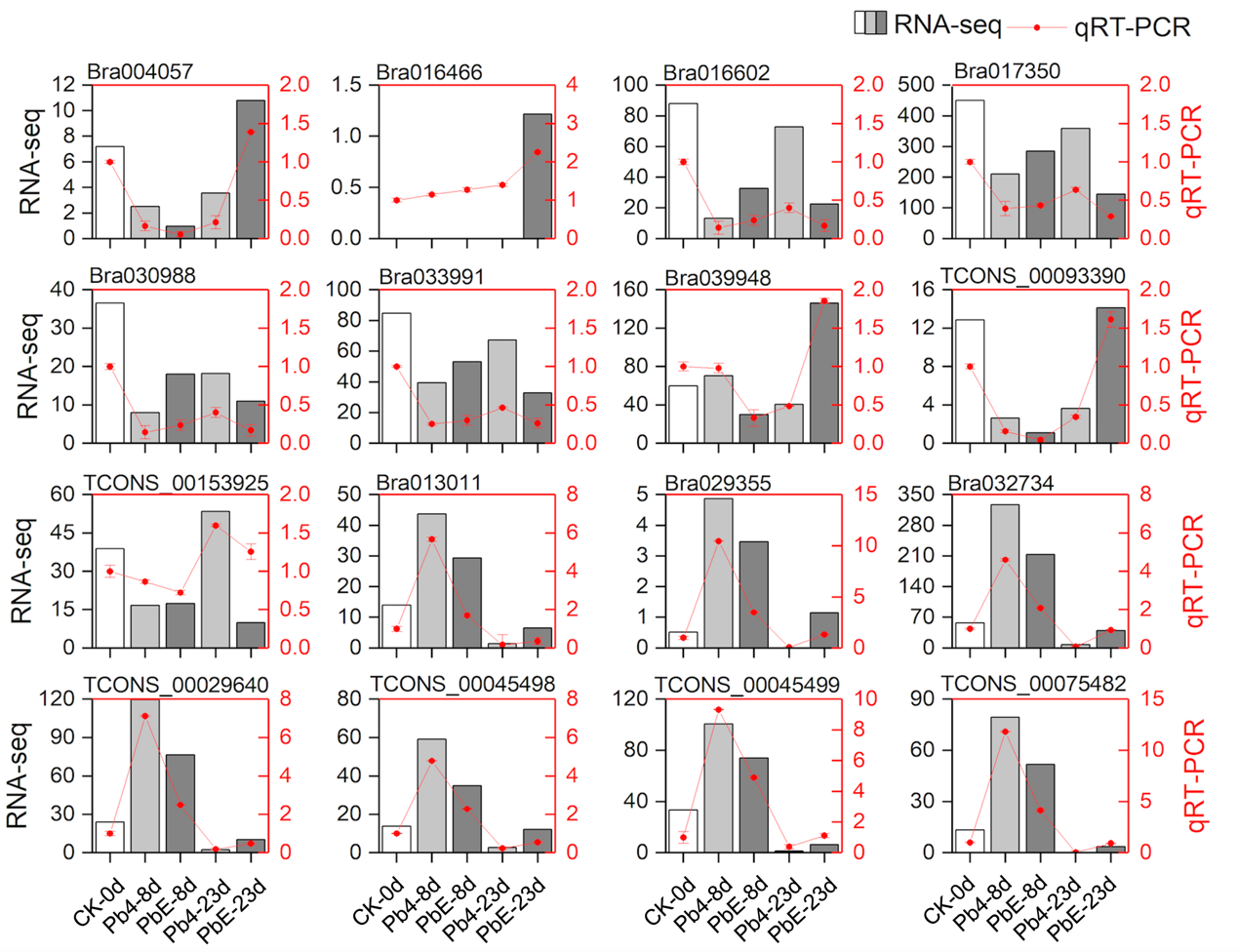


**Supplemental Figure 2. verification results by qRT-PCR.**

**
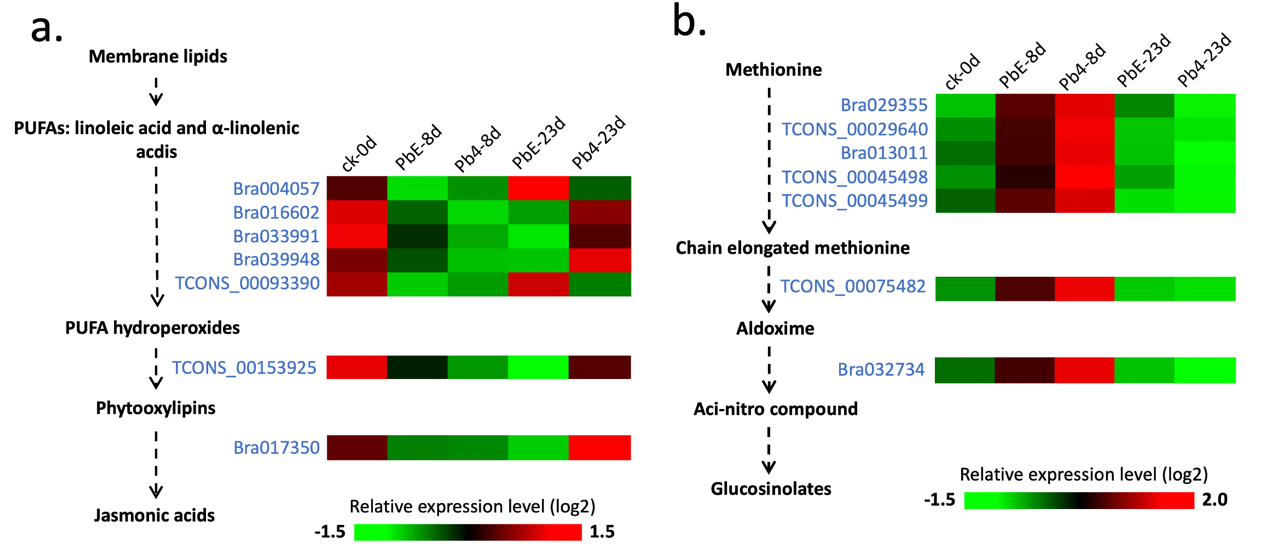
**

**Supplemental Figure 3.** Typical DEGs in the biosynthesis of the glucosinolate pathway **(a)**, and the linoleic acid and α-linolenic acid pathways **(b)**. The red and blue boxes represent the normalized relative expression levels.

**
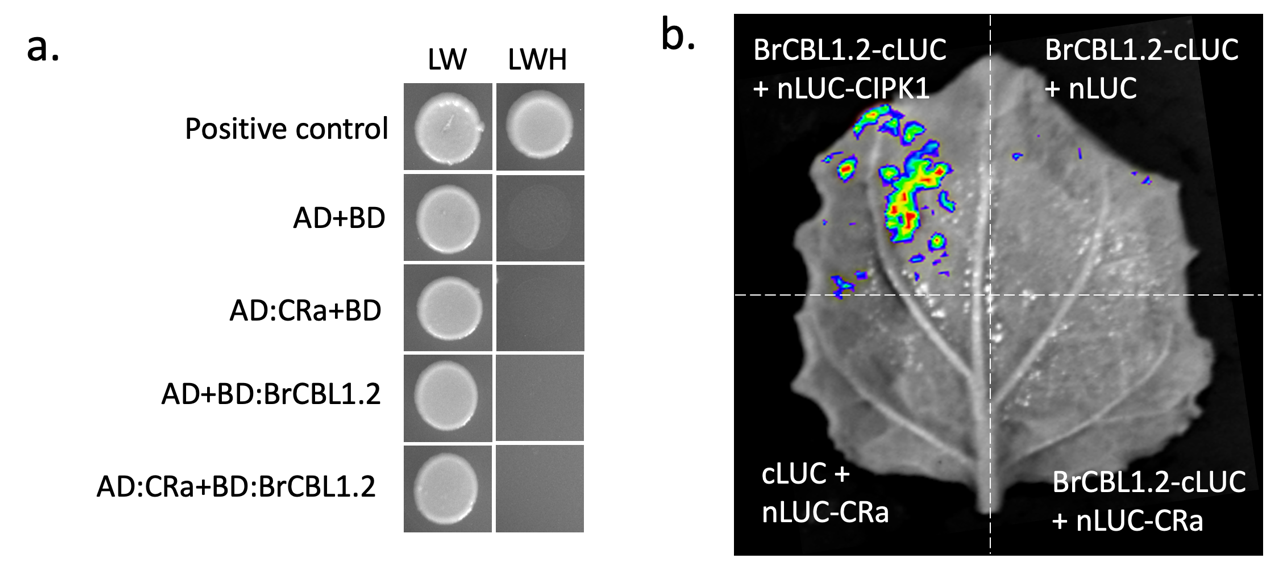
**

**Supplemental Figure 4. BraCBL1.2 interacts with AtCIPK1. (a)** CRa exhibits no interaction with BraCBL1.2 in yeast two hybrid assay. The combination of BD-BraCBL1.2 and AD-AtCIPK1 was taken as positive control. **(b)** Luciferase complementation assay shows there is no interaction between BraCBL1.2 and CRa. The combination of BraCBL1.2-cLUC and nLUC-AtCIPK1 was taken as positive control.

**
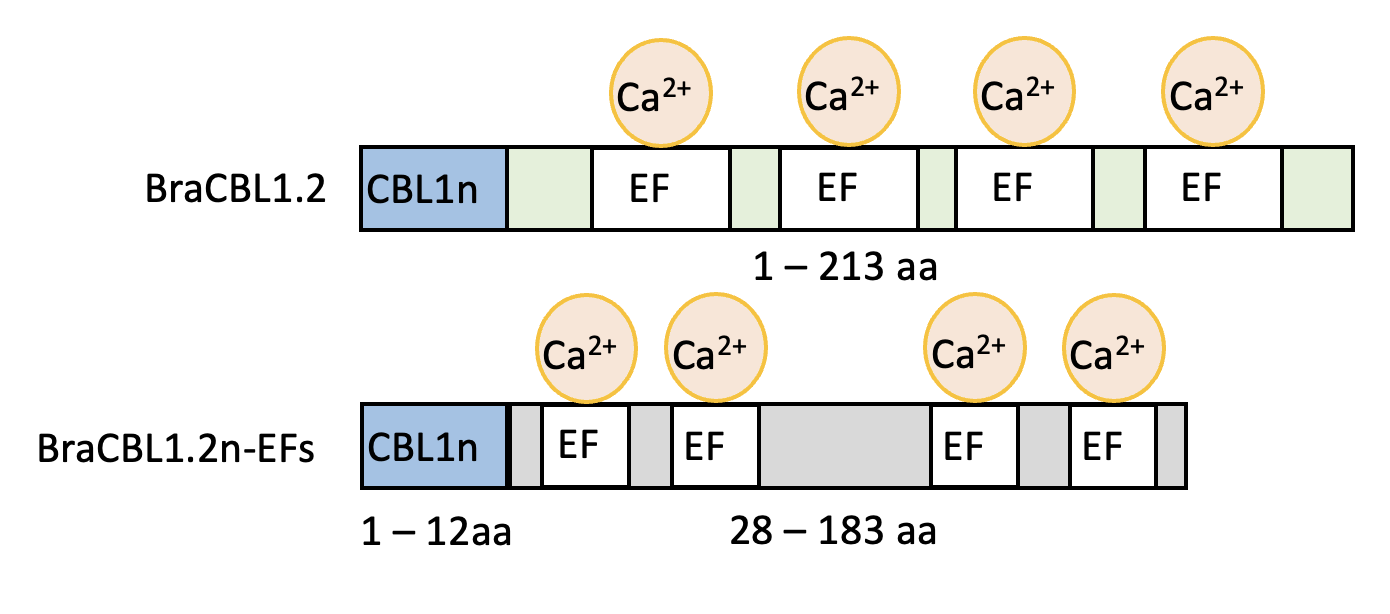
**

**Supplemental Figure 5. Functional domain of BraCBL1.2 and BraCBL1.2n-EFs.** The BraCBL1.2n-EFs has only the N-terminal 12 aa and 156 aa (from 28^th^ to 183^rd^ aa).

**
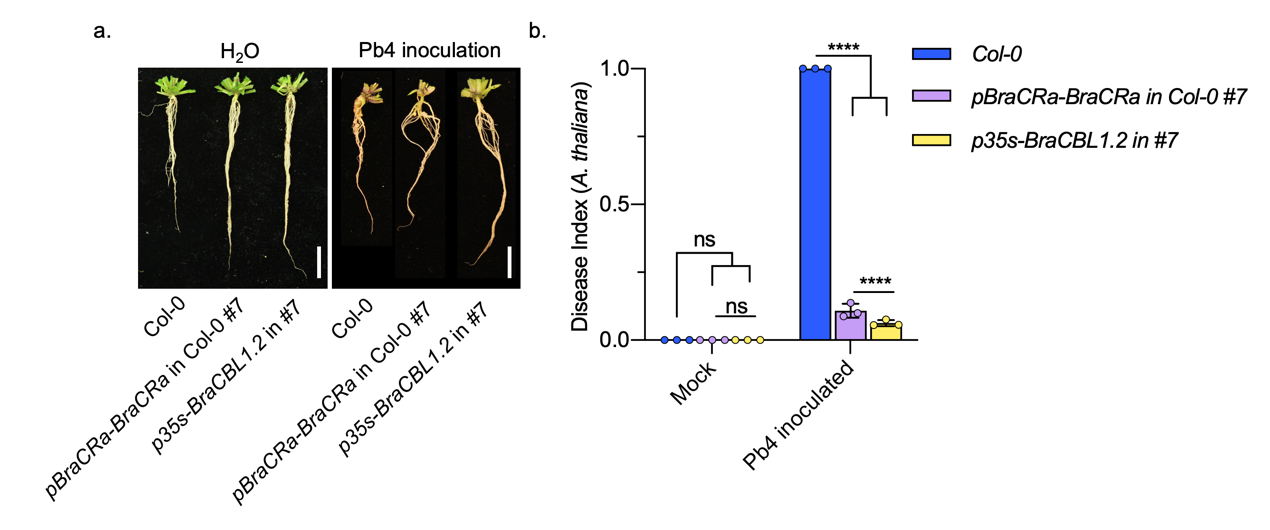
**

**Supplemental Figure 6. Overexpression of *BraCBL1.2* enhances Clubroot resistance in *pBraCRa:BraCRa* transgenic line #7.** Roots of 21 days old *Arabidopsis* were inoculated with sterile water (Mock), or Pb4. Gall formation was examined and photographed at 42 dpi as sign of clubroot disease. (a) phenotype of transgenic lines *pBraCRa:BraCRa* in Col-0 (#7) and *p35S:BraCBL1.2* in #7 after inoculation. Scale bar=1cm. (b) Disease index of transgenic lines. The graphs represent Disease index and error bars depict mean ± SD; Each dots represent one biological repeat (n = 15) ; *P* value was determined by One-way ANOVA analysis; NS, not significant; **** represents P less than 0.001.

**Supplemental Table 1. Number of up- or down-regulated DEGs in different comparison pairs.**

| **Pairs** | **DEG (Up)** | **DEG (Down)** |
| --- | --- | --- |
| **CK-0d-vs-Pb4-8d** | 849 | 981 |
| **CK-0d-vs-Pb4-23d** | 3510 | 1820 |
| **CK-0d-vs-PbE-8d** | 558 | 539 |
| **CK-0d-vs-PbE-23d** | 8331 | 3512 |
| **Pb4-8d-vs-Pb4-23d** | 2155 | 2830 |
| **PbE-8d-vs-Pb4-8d** | 55 | 74 |
| **PbE-8d-vs-PbE-23d** | 7144 | 3564 |
| **PbE-23d-vs-Pb4-23d** | 557 | 273 |

**Supplemental Table 2. Primer lists**

| **FIGURE 1b** |  |
| --- | --- |
| CRA FORWARD | ATGGATTTCTCTCTTTTCCTTACCATC |
| CRA REVERSE | ACATGAGGGAGTTTCCAGAGGATG |
| **FIGURE 2a** |  |
| CRA PROMOTER FORWARD | ﻿GTAAAACGACGGCCAGTGCCTTGTGAGGTGGGGAAAAGGATCAAT |
| CRA PROMOTER REVERSE | TTGTGATAACCTGTGTAAGCCAGTT |
| UIDA GENE FORWARD FOR PCRA | ﻿﻿AACTGGCTTACACAGGTTATCACAAATGTTACGTCCTGTAGAAACCCCA |
| UIDA GENE REVERSE | ﻿﻿AATTCGAGCTCGGTACCCGGTCATTGTTTGCCTCCCTGCTG |
| 35S PROMOTER FORWARD | ﻿GTAAAACGACGGCCAGTGCCAGCTTGCATGCCTGCAG |
| 35S PROMOTER REVERSE | ﻿GTCCCCCGTGTTCTCTCCA |
| UIDA GENE FORWARD FOR P35S | ﻿TGGAGAGAACACGGGGGACATGTTACGTCCTGTAGAAACCCCA |
| UIDA GENE REVERSE | ﻿﻿AATTCGAGCTCGGTACCCGGTCATTGTTTGCCTCCCTGCTG |
| **FIGURE 2c** |  |
| CRA PROMOTER FORWARD | ﻿GTAAAACGACGGCCAGTGCCTTGTGAGGTGGGGAAAAGGATCAAT |
| CRA PROMOTER REVERSE | TTGTGATAACCTGTGTAAGCCAGTT |
| CRA CDS FORWARD | AACTGGCTTACACAGGTTATCACAA﻿ATGGATTTCTCTCTTTTCCTTACCATC |
| CRA CDS REVERSE | ﻿ACATGAGGGAGTTTCCAGAGGATG |
| MVENUS FORWARD | ﻿TGGAAACTCCCTCATGTATGGTGAGCAAGGGCG |
| MVENUS REVERSE | ﻿﻿﻿AATTCGAGCTCGGTACCCGGTTACTTGTACAGCTCGTCCATGC |
| 35S PROMOTER FORWARD | ﻿GTAAAACGACGGCCAGTGCCAGCTTGCATGCCTGCAG |
| 35S PROMOTER REVERSE | ﻿GTCCCCCGTGTTCTCTCCA |
| MVENUS FORWARD | ﻿﻿TGGAGAGAACACGGGGGACATGGTGAGCAAGGGCG |
| MVENUS REVERSE | ﻿﻿﻿AATTCGAGCTCGGTACCCGGTTACTTGTACAGCTCGTCCATGC |
| **FIGURE 2d** |  |
| 35S PROMOTER FORWARD | ﻿GTAAAACGACGGCCAGTGCCAGCTTGCATGCCTGCAG |
| 35S PROMOTER REVERSE | ﻿GTCCCCCGTGTTCTCTCCA |
| CRA CDS FORWARD | AACTGGCTTACACAGGTTATCACAA﻿ATGGATTTCTCTCTTTTCCTTACCATC |
| CRA CDS REVERSE | ﻿ACATGAGGGAGTTTCCAGAGGATG |
| MVENUS FORWARD | ﻿TGGAAACTCCCTCATGT﻿ATGGTGAGCAAGGGCG |
| MVENUS REVERSE | ﻿﻿﻿AATTCGAGCTCGGTACCCGGTTACTTGTACAGCTCGTCCATGC |
| 35S PROMOTER FORWARD | ﻿GTAAAACGACGGCCAGTGCCAGCTTGCATGCCTGCAG |
| 35S PROMOTER REVERSE | ﻿GTCCCCCGTGTTCTCTCCA |
| MVENUS FORWARD | ﻿﻿TGGAGAGAACACGGGGGACATGGTGAGCAAGGGCG |
| MVENUS REVERSE | ﻿﻿﻿AATTCGAGCTCGGTACCCGGTTACTTGTACAGCTCGTCCATGC |
| **FIGURE 5a** |  |
| BRCBL1.2 PROMOTER FORWARD | ﻿﻿GTAAAACGACGGCCAGTGCCAGATCTCACCGCTAGAAACCATAC |
| BRCBL1.2 PROMOTER REVERSE | ﻿TTACAGAGATGCCAACAGACAAGATTC |
| UIDA GENE FORWARD FOR PCRA | ﻿GAATCTTGTCTGTTGGCATCTCTGTAAATGTTACGTCCTGTAGAAACCCCA |
| UIDA GENE REVERSE | ﻿﻿AATTCGAGCTCGGTACCCGGTCATTGTTTGCCTCCCTGCTG |
| **FIGURE 5 b ,c** |  |
| 35S PROMOTER FORWARD | ﻿GTAAAACGACGGCCAGTGCCAGCTTGCATGCCTGCAG |
| 35S PROMOTER REVERSE | ﻿GTCCCCCGTGTTCTCTCCA |
| BRCBL1.2 FORWARD | ﻿﻿TGGAGAGAACACGGGGGAC﻿ATGGGCTGCTTCCAATCAAAGGT |
| BRCBL1.2REVERSE | ﻿TGTGACAATCTCATCCACCTCCG |
| GFP FORWARD | ﻿CGGAGGTGGATGAGATTGTCACA﻿ATGAGTAAAGGAGAAGAACTTTTCACTG |
| GFP REVERSE | ﻿﻿AATTCGAGCTCGGTACCCGGTCA﻿TTTGTATAGTTCATCCATGCCATGTGT |
| ATCBL1 FORWARD | ﻿﻿TGGAGAGAACACGGGGGAC﻿ATGGGCTGCTTCCACTCAAAG |
| ATCBL1 REVERSE | ﻿TGTGGCAATCTCATCGACCTC |
| GFP FORWARD | ﻿GAGGTCGATGAGATTGCCACA﻿ATGAGTAAAGGAGAAGAACTTTTCACTG |
| GFP REVERSE | ﻿﻿AATTCGAGCTCGGTACCCGGTCA﻿TTTGTATAGTTCATCCATGCCATGTGT |
| **FIGURE 5 f** |  |
| 35S PROMOTER FORWARD | ﻿GTAAAACGACGGCCAGTGCCAGCTTGCATGCCTGCAG |
| 35S PROMOTER REVERSE | ﻿GTCCCCCGTGTTCTCTCCA |
| BRCBL1.2 FORWARD | ﻿﻿TGGAGAGAACACGGGGGAC﻿ATGGGCTGCTTCCAATCAAAGGT |
| BRCBL1.2REVERSE | ﻿AATTCGAGCTCGGTACCCGGTGTGACAATCTCATCCACCTCCG |
| 35S PROMOTER FORWARD | ﻿GTAAAACGACGGCCAGTGCCAGCTTGCATGCCTGCAG |
| 35S PROMOTER REVERSE | ﻿GTCCCCCGTGTTCTCTCCA |
| CRA CDS FORWARD | AACTGGCTTACACAGGTTATCACAA﻿ATGGATTTCTCTCTTTTCCTTACCATC |
| CRA CDS REVERSE | ﻿AATTCGAGCTCGGTACCCGGACATGAGGGAGTTTCCAGAGGATG |
| 35S PROMOTER FORWARD | ﻿GTAAAACGACGGCCAGTGCCAGCTTGCATGCCTGCAG |
| 35S PROMOTER REVERSE | ﻿GTCCCCCGTGTTCTCTCCA |
| BRCBL1.2n-EFs FORWARD | ﻿﻿TGGAGAGAACACGGGGGAC﻿﻿ATGGGCTGCTTCCAATCAAAGGTAGCAAGAGAATTC ﻿AGTGTGAGTGAGGTTGAAGC |
| BRCBL1.2n-EFs REVERSE | ﻿﻿AATTCGAGCTCGGTACCCGGTAGTGATGGGTTCTTGTTTACAAAATCGC |
|  |  |
| **SUPPLEMETNAL FIGURE 4a** |  |
| CRA CDS FORWARD FOR AD | ﻿ATACGACGTACCAGATTACGCTATGGATTTCTCTCTTTTCCTTACCATC |
| CRA CDS REVERSE FOR AD | ﻿﻿CTGCAGCTCGAGCTCGATCTAACATGAGGGAGTTTCCAGAGGATG |
| CRA CDS FORWARD FOR BD | GATCTCAGAGGAGGACCTGATGGATTTCTCTCTTTTCCTTACCATC |
| CRA CDS REVERSE FOR BD | ﻿GCGGCCGCTGCAGGTCGACCTAACATGAGGGAGTTTCCAGAGGATG |
| BRCBL1.2 FORWARD FOR BD | GATCTCAGAGGAGGACCTGATGGGCTGCTTCCAATCAAAGGT |
| BRCBL1.2REVERSE FOR BD | ﻿﻿GCGGCCGCTGCAGGTCGACCTATGTGACAATCTCATCCACCTCCG |
| ATCBL1 FORWARD FOR AD | ﻿ATACGACGTACCAGATTACGCTATGGGCTGCTTCCACTCAAAG |
| ATCBL1 REVERSE FOR AD | ﻿﻿﻿CTGCAGCTCGAGCTCGATCTATGTGGCAATCTCATCGACCTC |
| **SUPPLEMETNAL FIGURE 4b** |  |
| 35S PROMOTER FORWARD | ﻿GTAAAACGACGGCCAGTGCCAGCTTGCATGCCTGCAG |
| 35S PROMOTER REVERSE | ﻿GTCCCCCGTGTTCTCTCCA |
| BRCBL1.2 FORWARD | ﻿﻿TGGAGAGAACACGGGGGAC﻿ATGGGCTGCTTCCAATCAAAGGT |
| BRCBL1.2REVERSE | ﻿TGTGACAATCTCATCCACCTCCG |
| CLUC FORWARD | ﻿﻿CGGAGGTGGATGAGATTGTCACAATGTCCGGTTATGTAAACAATCCGG |
| CLUC REVERSE | ﻿CACGGCGATCTTTCCGC﻿﻿AATTCGAGCTCGGTACCCGG |
| NLUC FORWARD | ﻿﻿TGGAGAGAACACGGGGGAC﻿﻿GAAGACGCCAAAAACATAAAGAAAGGC |
| NLUC REVERSE | ﻿TCATCCATCCTTGTCAATCAAGGC |
| ATCIPK1 FORWARD | ﻿GCCTTGATTGACAAGGATGGATGA﻿ATGGTGAGAAGGCAAGAGGAG |
| ATCIPK1 REVERSE | ﻿﻿AATTCGAGCTCGGTACCCGGTCA﻿AGTTACTATCTCTTGCTCCGGC |
| CRA FORWARD | ﻿GCCTTGATTGACAAGGATGGATGAATGGATTTCTCTCTTTTCCTTACCATC |
| CRA REVERSE | ﻿﻿AATTCGAGCTCGGTACCCGGTCAACATGAGGGAGTTTCCAGAGGATG |
| qPCR primers |  |
| Bra016602F | CCCACCCGTCAGTAATCT |
| Bra016602R | GGTCTAGGAACGGTAAGAATA |
| Bra013011F | GCCGTCCAGTCAGTTGTA |
| Bra013011R | GCTTCTGTGGTGGAGTAAGT |
| Bra029355F | CTCGTCCTTGCTCATCTC |
| Bra029355R | AGCGTCGTGTCGAATACA |
| Bra032734F | CTGGTTACGACATCCCTG |
| Bra032734R | CCGAACGGTATAAACTCAT |
| Bra004057F | GGCTATGATGCTACCTCCTG |
| Bra004057R | ATGGCACTTAGTTGTCTGTTTG |
| Bra033991F | ACGATCCAGACTACGAAATG |
| Bra033991R | CTCCGTGAACTTGCTAAA |
| Bra039948F | CTTCGGGCAATACCCTTAC |
| Bra039948R | ACCGCTTGAACGCCTCTA |
| Bra016466F | AGAAGGGTGGTTGAAGACT |
| Bra016466R | TCATCAGGGATGGGTATT |
| Bra017350F | CGCCAGACCAAGTAAAGT |
| Bra017350R | CCATAGTCACCCAAGTAGAAA |
| Bra030988F | CTTAAACTCCTAATCGAA |
| Bra030988R | ATTCGGAGTACCAGGACT |
| TCONS_00093390F | GAATCTCAACCTGCTCAA |
| TCONS_00093390R | TTTCCTACGTCCAACAAT |
| TCONS_00153925F | CGTTTACCTCATTTCTTTAT |
| TCONS_00153925R | GAGTCGAGGAGAATTTTAGT |
| TCONS_00029640F | AGGCATTAGTAACGCTGTG |
| TCONS_00029640R | GAGGAAATCTGAGGGTCT |
| TCONS_00045498F | AGGTGAAAGAAGTGGGAATG |
| TCONS_00045498R | GCCAGGATACTCGGTTAG |
| TCONS_00045499F | CAACTATCCTCCAAATCG |
| TCONS_00045499R | AGCGTCGTGTCAAATACA |
| TCONS_00075482F | CGTTTCGTGTCGTTTGGT |
| TCONS_00075482R | ACGGCTGTCTTGTTTCCTTT |
